# Indirect Cost Analysis and Evaluation of Factors Affecting Indirect Costs in Patients Hospitalized with Acute Coronary Syndrome in Inpatient Wards of a Training and Research Hospital

**DOI:** 10.3390/healthcare14182896

**Published:** 2026-09-08

**Authors:** Özlem Karagöz, Uğur Karagöz, Büşra Tozduman, Melih Kaan Sözmen

**Affiliations:** 1 Department of Public Health, Faculty of Medicine, Atatürk Research and Training Hospital, Kâtip Çelebi University, Izmir 35620, Türkiye; busra.tozduman@ikc.edu.tr (B.T.); melihkaan.sozmen@ikc.edu.tr (M.K.S.); 2 Department of Cardiology, Faculty of Medicine, Atatürk Research and Training Hospital, Kâtip Çelebi University, Izmir 35620, Türkiye; ugur.karagoz@ikc.edu.tr

**Keywords:** acute coronary syndrome, indirect cost, productivity loss, cost of illness, health economics

## Abstract

**Highlights:**

**What are the main findings?**

- The median one-month total indirect cost per hospitalized patient with acute coronary syndrome (ACS) was 1007 Int$. Absenteeism was the dominant component within the actively employed subgroup, whereas household production loss accounted for the entire cost in the 82 patients outside the labor force.
- In multivariate generalized linear modeling with a Gamma distribution and log link, occupational status and female sex were the variables most strongly and independently associated with the indirect economic burden. After adjustment for occupational group and sex, clinical severity and quality-of-life scores did not reach statistical significance.

**What are the implications of the main findings?**

- The short-term indirect economic burden of acute coronary syndrome extends beyond direct medical expenses, highlighting measurable immediate costs at the patient and household levels.
- Restricting outcome assessment in ACS to clinical endpoints omits a measurable component of the early post-discharge burden, which falls on both paid and unpaid work.

**Abstract:**

**Background/Objectives**: This study aims to determine the indirect costs associated with the disease process in patients hospitalized for acute coronary syndrome and to analyze the sociodemographic and clinical factors influencing these costs. **Methods:** This prospective, single-center, observational cost-of-illness study evaluated 146 ACS patients at a tertiary cardiology clinic over an index episode extending from admission to 30 days after discharge, from a patient and household perspective that excludes direct medical costs. Excluding six patients (one death, five early retirements), 140 patients were analyzed. Productivity losses were measured with the iMTA Productivity Cost Questionnaire and valued using the Human Capital Approach; SCORE2, GRACE and MIDAS scores were also recorded. Costs are reported in 2025 international dollars (Int$, OECD PPP factor 16.20). Factors associated with total indirect cost were examined with a Gamma generalized linear model with a log link, containing five prespecified covariates: sex, occupational group, age, GRACE and MIDAS. **Results:** The mean age was 61 ± 12 years, and 69.9% of the cohort were male. The median total indirect cost per patient was 1007 Int$. In the actively employed subgroup, the median absenteeism cost was 1450 Int$, while the median household production loss for the entire cohort was 340 Int$. The multivariate Gamma GLM identified occupational status (*p* < 0.001) and female sex (*p* = 0.004) as variables independently associated with total indirect costs, whereas age and clinical/prognostic scores (GRACE, MIDAS) lacked statistical significance. Compared to patients outside the labor force, total costs were 4.8 and 4.1 times higher for white-collar and blue-collar workers, respectively. **Conclusions:** ACS imposes a significant short-term indirect cost burden at the patient and household levels, driven by absenteeism among actively employed patients and by household production losses among those outside the labor force. These findings indicate that the early post-discharge economic burden is patterned primarily by labor force position and by the distribution of unpaid domestic work rather than by clinical severity.

## 1. Introduction

Cardiovascular diseases (CVDs) remain the leading cause of mortality and morbidity worldwide, accounting for an estimated 18.6 million deaths in 2019 [1]. According to the Global Burden of Disease (GBD) 2023 report, ischemic heart disease ranks first among the causes of premature mortality and disability-adjusted life year (DALY) loss [2]. Acute Coronary Syndromes (ACSs), the most emergent and costly clinical manifestation of these ischemic conditions, are characterized by the sudden interruption of coronary blood flow and encompass Unstable Angina Pectoris (UAP), Non-ST-Segment Elevation Myocardial Infarction (NSTEMI), and ST-Segment Elevation Myocardial Infarction (STEMI) [3]. In Türkiye, circulatory system diseases account for 36.0% of total mortality, of which 42.9% is directly attributed to ischemic heart diseases [4]. Given that the working-age population (under 65 years of age) accounts for approximately 47% of all ACS patients, the disease extends beyond a purely medical condition, imposing profound consequences on households, employers, and the national economy [5,6].

In recent years, advances in medical technology and percutaneous coronary interventions (PCIs) have reduced ACS-related in-hospital mortality and improved survival has, however produced a larger population living with reduced functional capacity, shifting the burden from acute mortality towards long-term morbidity and productivity loss [7]. The overall economic burden of a disease comprises direct, indirect, and intangible costs [8]. Cost-of-illness analyses in the literature predominantly focus on direct medical costs, encompassing hospitalizations, surgical interventions, and pharmaceutical expenses [9,10]. However, indirect costs resulting from ACS-related work incapacity, disability, or premature mortality among the working-age population constitute a frequently overlooked yet substantial portion of the total economic burden [11,12].

Patients with Acute Coronary Syndrome (ACS) experience a complex and substantial loss of productivity, extending from hospitalization and post-discharge convalescence at home to periods of diminished functional capacity after returning to work [13]. Indirect costs of this kind are commonly measured with instruments such as the iMTA Productivity Cost Questionnaire (iPCQ), which provides a standardized assessment of morbidity-related losses (absenteeism, presenteeism, and unpaid labor loss) and is recognized as an international reference [14,15]. Although clinical treatment protocols for the management of ACS in Türkiye align with international standards, evidence on its socioeconomic consequences remains limited. Analyses conducted from a General Health Insurance (GSS) perspective predominantly define costs as cash outflows from the reimbursement institution’s budget and do not incorporate productivity losses [11].

In this context, the primary objective of this study is to determine from a patient and household perspective, on a one-month basis, the indirect costs incurred by the disease process among patients hospitalized with a diagnosis of ACS at a tertiary care center, and to analyze of the factors associated with these costs.

## 2. Materials and Methods

### 2.1. Study Design and Sample

**Figure 1:**
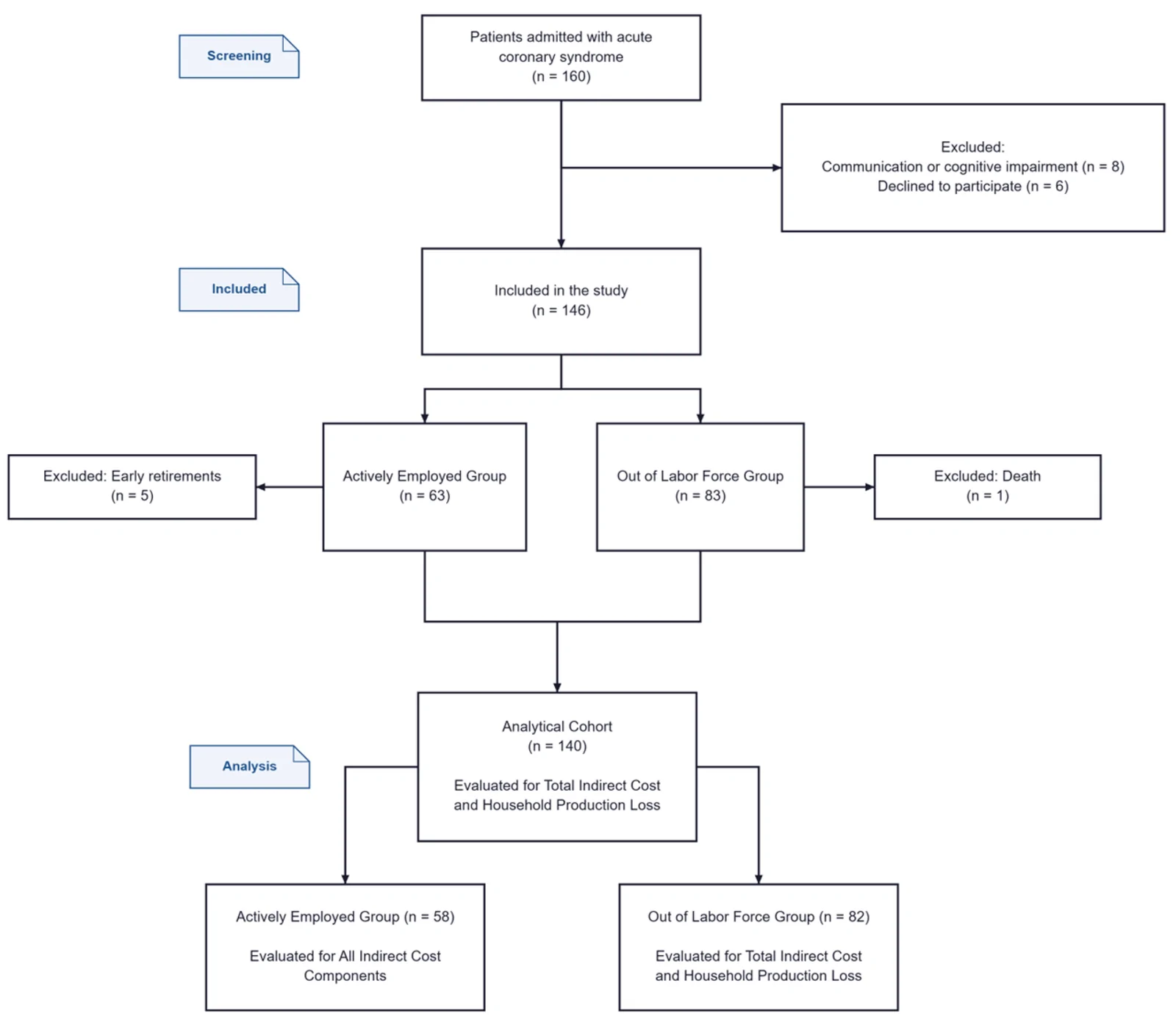
Flow diagram of study participants, exclusions, and analytical subgroups.

This prospective, single-center, observational cost-of-illness study was conducted at the Department of Cardiology of Izmir Katip Celebi University Atatürk Training and Research Hospital to determine the indirect costs associated with the disease process in patients who experienced ACS. Data were collected over a six-month period from March to August 2025. The study sample size was calculated as a minimum of 116 based on a regression coefficient (beta = −0.594) from a reference study investigating indirect costs following myocardial infarction [16], with 90% statistical power and a significance level of alpha = 0.05. During the study period, 160 patients admitted with acute coronary syndrome were screened for eligibility. Eight were excluded because of communication or cognitive impairment, and six declined to participate, leaving 146 patients who provided informed consent and were enrolled (participation rate 96.1% of those eligible). The flow of participants, including subsequent exclusions and analytical subgroups, is presented in Figure 1. The economic analysis was conducted from a patient and household perspective, capturing productivity losses borne directly by the patient and household while excluding all direct medical costs (e.g., hospitalization fees, interventional procedures, and pharmaceutical expenses), employer-side costs and health-system burdens.

### 2.2. Data Collection Tools and Clinical Evaluation

Baseline sociodemographic and clinical data were obtained through face-to-face interviews and from Hospital Information Management System (HIMS) records during hospitalization. The costs incurred during the index episode, defined as the period from hospital admission to 30 days after discharge, were analyzed. The iMTA Productivity Cost Questionnaire (iPCQ) was administered via telephone one month after discharge, utilizing a four-week recall period covering the post-discharge month. The length of the index admission was obtained from hospital records. The diagnosis of acute coronary syndrome and its classification into STEMI, NSTEMI and unstable angina were established according to the 2023 European Society of Cardiology guidelines for the management of acute coronary syndromes [3]. The clinical risk profiles of the patients (including diabetes, renal failure, and lipid levels) were determined in accordance with the 2021 European Society of Cardiology (ESC) guideline standards [17]. SCORE2 was used to determine the 10-year risk of cardiovascular events in patients aged over 40 years, whereas the GRACE score was used to evaluate the prognostic risk of ACS, with a GRACE score > 140 defined as the high-risk group [3]. Quality of life following the cardiovascular event was measured using the Myocardial Infarction Dimensional Assessment Scale (MIDAS), which consists of 35 items across seven dimensions; higher scores indicate poorer health status [18].

### 2.3. Calculation of Indirect Costs

The iMTA Productivity Cost Questionnaire (iPCQ), consisting of three primary modules (absenteeism, presenteeism, and unpaid labor), was employed to objectively measure the productivity losses generated by ACS. Productivity loss due to presenteeism was assessed using a 0–10 Verbal Numerical Rating Scale (VNRS) for compatibility with telephone interviews. The Human Capital Approach was adopted for economic calculations. Each cost component was incurred over the interval in which it is defined. For patients in paid employment, absenteeism was incurred over the entire episode; the days of the index admission, retrieved from hospital records, were added to the days of post-discharge absence reported in the iPCQ, as both represent paid work time lost to the event. Presenteeism and household production loss were measured only during the post-discharge month, since these are not defined during an inpatient stay. All components were valued from the patients’ self-reported monthly income or the shadow price described below, and all estimates were structured on a one-month basis rather than through annual extrapolation. Total indirect cost was defined as the sum of household production loss, absenteeism cost and presenteeism cost. For patients outside the labor force, absenteeism and presenteeism are undefined and total indirect cost equals household production loss; for actively employed patients it is the sum of all three components.

The unpaid labor module was administered directly to patients via telephone interviews using a four-week recall period. Patients reported the number of days on which domestic activities (e.g., cleaning, shopping, childcare) were reduced due to health reasons, along with the average daily hours of assistance received from another person on those days. Household production loss was valued using the replacement cost method, applying a single shadow price to these assistance hours: the 2025 Turkish national net minimum wage of TRY 22,104.67 per month, corresponding to TRY 98.24 per hour when divided by the standard monthly working time of 225 h used in Turkish wage calculations [19]. While the questionnaire captured whether assistance was received, it did not record the provider’s relationship to the patient. All raw cost data were collected in local currency (Turkish Lira, TRY). Wages were monetized based on patients’ self-reported monthly net income at the time of the interview, whereas assistance hours were valued using the single 2025 shadow price outlined above. All costs are reported in nominal terms, reflecting the price levels prevailing at the time of each patient interview. Because individual interview dates were not retained in the analytical dataset, values could not be deflated to a single common base date, and the estimates are consequently not standardized to a single reference month. Rather, the estimates represent nominal 2025 values pooled across the data collection window between March and August 2025. To quantify the potential uncertainty, Appendix A Table A2 presents a sensitivity analysis by rebasing all observations to both the earliest and latest months of this collection window.

Primary results are presented in 2025 TRY, with 2025 international dollars (Int$) provided concurrently to facilitate cross-national comparison. Currency conversion applied the OECD Purchasing Power Parity (PPP) factor for gross domestic product in Türkiye for 2025 (16.20 TRY per Int$), retrieved from the OECD Purchasing Power Parities and Exchange Rates dataset [20]. The series is updated annually to reflect domestic price developments, with the corresponding factor for 2024 set at 11.55 TRY per international dollar. The implied price level index relative to the nominal exchange rate was 0.35 in 2024 and 0.41 in 2025, remaining far below unity in both years and confirming that this metric reflects domestic purchasing power rather than short-term market exchange rate fluctuations. Values in subsequent comparative tables are reported exclusively in Int$, with corresponding TRY figures obtainable by applying this conversion factor.

### 2.4. Statistical Analysis

#### 2.4.1. Analytical Cohort and Denominators

Three distinct features of the dataset determine the denominators used throughout the analyses.

First, six of the 146 enrolled patients were excluded from the cost analyses, yielding an analytical cohort of 140: one patient outside the labor force who died during follow-up, and five actively employed patients who left work or took early retirement because of the index event. These cases are incompatible with the short-term measurement structure of the iPCQ, since incorporating a lifetime income loss into a one-month horizon would introduce severe methodological heterogeneity and extreme leverage. They are nevertheless retained in the descriptive statistics for the enrolled sample (*n* = 146).

Second, some variables are structurally undefined in parts of the cohort. Absenteeism and presenteeism do not apply to patients outside the labor force, who have no paid work time to forgo; these two components were therefore evaluated only in the 58 patients who were in salaried employment before the index event and are reported as not applicable elsewhere. Likewise, SCORE2 is defined only from the age of 40 years, which excludes six patients and gives denominators of 135 in the cost cohort and 55 in the employed subgroup. Neither situation represents missing data.

Third, within these denominators, data collection was complete. Self-reported monthly household income, MIDAS and GRACE scores, and all cost components contained no missing values, so no imputation was required. All analyses are therefore complete-case analyses on the denominators stated in each table.

#### 2.4.2. Descriptive Statistics

Data were analyzed using IBM SPSS Statistics for Windows, version 27.0 (IBM Corp., Armonk, NY, USA). Normality was assessed with the Shapiro–Wilk test. Age is reported as mean ± standard deviation, and cost variables, which were not normally distributed, as median (25th–75th percentile). Because monetary variables were skewed, bias-corrected and accelerated bootstrap 95% confidence intervals based on 1000 resamples were computed for mean costs. Percentiles were calculated with the weighted-average definition, and bootstrap resampling used the Mersenne Twister generator with a fixed seed to ensure reproducibility.

#### 2.4.3. Bivariate Comparisons

Because the cost components contained a high proportion of zero values and the employed subgroup was small, groups were compared with non-parametric tests: the Mann–Whitney U test for two groups and the Kruskal–Wallis test for three or more, with Dunn’s test and Bonferroni adjustment for pairwise comparisons where the overall test was significant. These comparisons were exploratory and were not adjusted for multiple testing across variables.

#### 2.4.4. Multivariable Model

Total indirect cost was strictly positive (minimum 85 Int$; no zero values) and right-skewed (skewness = 1.55, kurtosis = 1.90). Zero values occurred only in the individual components, which were not modeled separately: 1 of 140 patients for household production loss and 27 of 58 employed patients for presenteeism. Total indirect cost was therefore modeled using a generalized linear model with a Gamma distribution and a log-link function, fitted with Huber–White robust standard errors. Results are presented as cost ratios (Exp[B]) with 95% confidence intervals. The overall significance of the model was assessed with the omnibus test and the effect of each covariate with the Wald chi-square test.

The five covariates were specified a priori on conceptual grounds; no stepwise or automated selection was used. Occupational status was entered as a stratifying variable rather than an etiological predictor: because absenteeism and presenteeism are valued from wages, labor force position partly determines the cost formula itself, and its coefficient therefore describes how cost varies across strata rather than an independent effect of occupation on cost. The model estimated seven regression parameters (intercept, one contrast for sex, two contrasts for occupational status, and three continuous covariates) together with a scale parameter, giving approximately 20 observations per estimated parameter. Multicollinearity was evaluated prior to model fitting using variance inflation factors (VIFs) and tolerance values derived from an equivalent ordinary least squares specification; all VIFs remained below 2 (maximum: 1.978; minimum tolerance: 0.506). Continuous variables (age, MIDAS, and GRACE) were mean-centered to ensure a clinically interpretable intercept.

#### 2.4.5. Model Diagnostics and Sensitivity Analysis

The variance function was examined with a modified Park test: the natural logarithm of the squared raw residuals from the fitted model was regressed on the natural logarithm of the predicted means, so that the slope estimates the power parameter λ in Var$\left(y\right)\propto \;\mu^{\lambda }$. The estimated power was 1.71 (95% CI: 1.31–2.12). This confidence interval includes the value of 2 corresponding to the Gamma distribution family, indicating that a Gamma variance structure is consistent with the observed data. Because this diagnostic does not definitively distinguish the Gamma family from adjacent variance specifications, robust standard errors were used so that inference remains valid under possible misspecification of the variance function.

Influential observations were identified using standardized deviance residuals, with an absolute value of 3 taken as the threshold for an extreme value, and Cook’s distance, with the sample-size-adjusted threshold of 4/*n*. The robustness of the model was then examined in two ways. First, the model was refitted after excluding the observations exceeding the Cook’s distance threshold, in order to establish whether the primary estimates depended on a small number of influential patients. Second, an alternative specification was fitted in which the natural logarithm of total indirect cost was regressed on the same five covariates by ordinary least squares, in order to establish whether the findings were specific to the Gamma-log formulation. Estimates from both analyses are reported in Section 3.6.

## 3. Results

### 3.1. Baseline Characteristics and General Profile of the Patients

Of the total 146 patients included in the study, 69.9% (*n* = 102) were male, and the mean age of the overall cohort was determined to be 61 ± 12 years. While 83.6% of the patients were covered by General Health Insurance, 64.4% had an educational attainment of primary school or lower (Table 1). Of the study cohort, 43.2% (*n* = 63) were in active salaried employment, whereas 56.8% (*n* = 83) were outside the labor force. This ‘out of labor force’ cohort specifically consisted of retirees (*n* = 54), homemakers (*n* = 25), and unemployed individuals (*n* = 4). Upon examination of the clinical characteristics, the most common risk factors were smoking (63.7%), diabetes (32.9%), and a history of previous coronary artery disease (28.1%); STEMI was the most common presentation (54.8%). Among patients included in the SCORE2 risk classification, 53.6% fell into the “very high” risk category, and 41.1% of the patients were classified as high-risk according to the GRACE risk score. The median MIDAS total score, reflecting the disease-specific quality of life, was found to be 19.3; whereas the median total GRACE score, a prognostic risk indicator, was 132.

### 3.2. General Distribution of One-Month Indirect Costs

One-month total indirect cost analyses related to the disease were calculated based on 140 patients, excluding those incompatible with the short-term measurement dynamics of the iPCQ (those who died or retired early due to permanent loss of labor). In the overall cohort, the median one-month household production loss was identified as 340 Int$, and the calculated median total one-month indirect cost was 1007 Int$ (Table 2). Because absenteeism and presenteeism are undefined for patients outside the labor force, these components were analyzed exclusively in the subgroup of actively salaried patients (*n* = 58), in which the median one-month absenteeism cost was 1450 Int$ and the median one-month presenteeism cost was 96 Int$.

### 3.3. Comparison of One-Month Indirect Costs by Sociodemographic and Clinical Factors

The analysis of one-month indirect costs according to sociodemographic characteristics revealed that female patients demonstrated significantly higher one-month absenteeism costs (*p* = 0.041) and household production losses (*p* < 0.001) than male patients. The median one-month total indirect cost in the salaried employee group (2113 Int$) was significantly higher than that of the group outside the labor force (679 Int$) (*p* < 0.001) (Table 3). Detailed analysis by occupational group showed that median total indirect cost was higher among white-collar (2407 Int$) and blue-collar (1901 Int$) workers than among patients outside the labor force (*p* < 0.001) (Table 4).

When evaluating the impact of household structure on one-month costs, the median total one-month indirect cost was lower among patients living in a nuclear family with spousal support (679 Int$) than among those living alone (1358 Int$) (*p* = 0.022) (Table 5). Although no statistically significant differences were observed across educational attainment levels regarding overall one-month total indirect costs or one-month absenteeism costs (*p* > 0.05), one-month household production loss was significantly higher among patients with a primary school education or lower compared to those with higher educational attainment (*p* = 0.003) (Appendix A Table A1).

Upon examination of clinical comorbidities and risk factors, one-month total indirect costs were significantly higher in patients without diabetes or a history of prior coronary artery disease (*p* < 0.05). Absenteeism cost was significantly lower in patients with renal failure compared to those without (561 vs. 1564 Int$; *p* = 0.022). Furthermore, one-month household production loss was higher in non-smokers (*p* = 0.019) whereas absenteeism cost was higher among active smokers (*p* = 0.031); total indirect cost was significantly higher in smokers (1075 vs. 819 Int$; *p* = 0.007) (Table 3). The same direction was observed for the GRACE classification, in both absenteeism cost (1646 vs. 840 Int$ in the normal- and high-risk groups; *p* = 0.023) and total indirect cost (*p* = 0.001); for SCORE2 the very-high-risk group carried the lowest total cost (*p* = 0.005). However, when the analysis was restricted to patients eligible for these outcomes (the actively employed subgroup), no significant differences remained in one-month absenteeism costs based on the presence of diabetes (*p* = 0.097), prior coronary artery disease (*p* = 0.270), or SCORE2 risk groups (*p* = 0.372) (Table 6). No statistically significant difference in one-month presenteeism cost was observed for any sociodemographic or clinical variable examined (Table 3, Table 4, Table 5 and Table 6 and Appendix A Table A1); within the actively employed subgroup, presenteeism was correlated only with patient age (rho = −0.294; *p* < 0.05). This reflects both the restriction of the analysis to 58 patients and the high proportion of zero values in this component (27/58).

### 3.4. Correlations Between Variables

Table 7 presents the Spearman correlation coefficients between continuous variables and cost parameters. Increasing patient age was positively correlated with one-month household production loss (rho = 0.233; *p* < 0.01) but inversely correlated with one-month absenteeism (rho = −0.344; *p* < 0.01), presenteeism (rho = −0.294; *p* < 0.05), and total indirect cost (rho = −0.378; *p* < 0.01). One-month household income showed a positive correlation with absenteeism cost (rho = 0.506; *p* < 0.01) and total indirect cost (rho = 0.354; *p* < 0.01), but was inversely correlated with household production loss (rho = −0.257; *p* < 0.01). The MIDAS score exhibited a weak positive correlation exclusively with household production loss (rho = 0.198; *p* < 0.05). Finally, the GRACE score demonstrated a significant inverse correlation with total indirect cost (rho = −0.288; *p* < 0.01). When absenteeism and presenteeism outcomes were restricted to the actively employed subgroup (*n* = 58), neither the MIDAS score nor the GRACE score showed any significant correlation with these paid-productivity costs.

### 3.5. Multivariate Analysis of Total One-Month Indirect Costs

The model was significant against the intercept-only model (likelihood ratio χ^2^ = 96.392, df = 6, *p* < 0.001), and the scaled Pearson χ^2^/df ratio of 0.907 indicated adequate fit. In the generalized linear model with a Gamma distribution and log link, occupational group (Wald χ^2^ = 89.051; *p* < 0.001) and female sex (Wald χ^2^ = 8.322; *p* = 0.004) were the only covariates independently associated with total one-month indirect cost; the group outside the labor force served as the reference category. Patient age (*p* = 0.701), the GRACE score (*p* = 0.508) and the MIDAS total score (*p* = 0.094) showed no independent association once occupational group and sex were held constant (Table 8).

Given the log link specification, exponentiated coefficients are interpreted as multiplicative cost ratios applied to the baseline intercept. The intercept, 561.77 Int$ (95% CI 460.93–684.67), is the expected one-month indirect cost of a male patient outside the labor force at the cohort mean age, GRACE score and MIDAS score. Relative to that reference, the cost ratio was 4.785 (95% CI 3.104–7.375) for white-collar workers and 4.109 (95% CI 2.999–5.629) for blue-collar workers, and 1.465 (95% CI 1.130–1.900) for women. Applying these ratios to the intercept gives expected one-month indirect costs of approximately 2690 Int$ for a white-collar man, 2310 Int$ for a blue-collar man and 560 Int$ for a man outside the labor force, with each value approximately 1.5 times higher for women (3940, 3380 and 820 Int$, respectively). These values are model-predicted means calculated at cohort-average covariate values and are not directly comparable with the observed medians in Table 3 and Table 4.

The Wald statistics indicate that labor force position had a stronger association with costs than sex, whereas clinical severity and disease-specific quality of life showed no independent association after controlling for both variables.

### 3.6. Model Diagnostics and Sensitivity Analysis

Influence diagnostics and sensitivity analyses were conducted to evaluate the robustness of the primary findings. Standardized deviance residuals revealed no extreme values exceeding an absolute threshold of 3, with values ranging from −2.57 to 1.84. Although five observations exhibited a Cook’s distance exceeding the conventional sample-size-adjusted threshold (4/*n* = 0.0286; maximum value: 0.160), none showed evidence of severe model distortion. A sensitivity analysis excluding these five influential observations yielded results consistent with the primary model: the cost ratio was 5.090 (95% CI 3.826–6.771) for white-collar and 4.399 (95% CI 3.264–5.929) for blue-collar workers relative to the out-of-labor-force reference, and 1.480 (95% CI 1.143–1.915) for female sex (*p* = 0.003), demonstrating stability against the primary model estimates of 4.785, 4.109, and 1.465, respectively. Notably, the MIDAS total score reached statistical significance in this restricted cohort (Exp[B] = 1.006, *p* = 0.020). This association is therefore not robust, as it emerges only when five observations are removed and is not claimed as a finding of this study. Furthermore, an alternative log-transformed linear specification regressing the natural logarithm of total cost on the same covariates preserved the magnitude, direction, and significance of the primary predictors, yielding cost ratios (Exp[B]) of 5.063 (95% CI 3.126–8.199) for white-collar workers, 4.209 (95% CI 3.026–5.856) for blue-collar workers, and 1.493 (95% CI 1.076–2.071) for female sex (*p* = 0.017). Taken together, these checks indicate that the associations with labor force position and sex are not produced by a small number of influential observations and are not specific to the Gamma-log formulation.

## 4. Discussion

This research quantifies the indirect economic burden of ACS patients in Türkiye across the dimensions of absenteeism, presenteeism, and household production loss, utilizing a multivariate modeling approach based on the iPCQ methodology, which was developed by the Institute for Medical Technology Assessment in the Netherlands and is considered the gold standard in the international literature. Upon examining the demographic structure of the study cohort comprising 146 patients included in our research, it is observed that the mean age is 61 ± 12 years, and 43.2% of the patients are actively engaged in salaried employment. This situation demonstrates that ACS is not an issue limited to the geriatric population, but rather one that directly impacts active human capital responsible for generating added value and providing for household livelihoods. The median total indirect cost per patient reaching 1007 Int$ in our study highlights the substantial socioeconomic burden of cardiovascular events beyond their clinical implications. When evaluated in conjunction with the existing literature, the associations between indirect cost and sociodemographic characteristics, occupational status, household structure and prognostic scores are examined below, including those that run counter to the direction expected for direct costs.

### 4.1. Economic Dimensions of Productivity Losses, Absenteeism, and Presenteeism Dynamics

The per-patient indirect cost and productivity loss observed in this cohort indicate that the burden of ACS extends beyond direct medical expenditure to the patient’s own paid and unpaid work. In the analyses conducted within the framework of the Human Capital Approach, the median absenteeism cost was calculated as 1450 Int$ and the presenteeism cost as 96 Int$ in the subgroup of 58 actively salaried patients. Considering the minimum wage and daily gross earnings parameters during the study period, these data demonstrate that a single acute coronary event results in a significant economic loss in terms of an employee’s labor capacity and productivity.

In the literature, it is emphasized that absenteeism resulting from ACS constitutes the primary and often the largest component of the total economic cost. Comprehensive cost-effectiveness studies conducted by Pirhonen et al. in Sweden demonstrate that preventing labor losses—particularly within the active population under 65 years of age—represents the most significant economic gain afforded by person-centered care [21]. In a retrospective study conducted by Zhao and Winget using commercial insurance databases, the average annual absenteeism cost in ACS cases was reported to be $9733 in the group receiving medical management alone [22]. Recent evidence indicates that although modern percutaneous revascularisation and early rehabilitation shorten the time to return to work in younger patients [23], the overall macroeconomic indirect cost burden driven by premature mortality and morbidity remains remarkably substantial across the continent [11].

The analysis by Song et al. in the United States among employees at high cardiovascular risk highlights the financial implications of labor loss. During the first year after a cardiovascular event, the average monthly absenteeism cost per patient was $698, and short-term disability cost was $281, bringing the total monthly cost to $847. Myocardial infarction was among the clinical presentations associated with the highest absenteeism costs at the end of the first year [24]. The median labor loss cost of 1450 Int$ in our salaried employee group is consistent with the substantial economic burden reported in previous studies. This finding indicates that productivity loss continues after discharge during return to work and functional recovery.

In our study, total indirect costs were significantly higher among salaried employees than among those outside the labor force. This finding is consistent with the Human Capital Approach, as calculations based on market wages show that disease-related productivity losses among active workers can generate substantial economic losses for both national income and employers [25]. A numerically higher median presenteeism cost was observed among manual workers than among office-based workers, although this difference did not reach statistical significance and the office-based stratum comprised only nine patients. This pattern is nevertheless consistent with the clinical effects of ACS, which can limit exertional capacity and physical endurance and may therefore have a greater impact on physically demanding occupations [26].

### 4.2. Economic Value of Household Production Loss and Differences by Sex

The value of lost household production and unpaid domestic labor due to the patient’s incapacity during the post-discharge recovery period is a component often overlooked in indirect cost calculations. Defined in the literature as “invisible labor,” it represents a component with a substantial aggregate economic dimension [21]. Across the study cohort, the median economic value of household production loss during illness and recovery was 340 Int$, with 25% of patients incurring more than 679 Int$ per month. These findings are consistent with data in the international literature. In a study conducted by Tumanan-Mendoza et al. in the Philippines, it was emphasized that the full-time labor loss of at least one caregiver during the hospitalization period, combined with the patient’s individual productivity loss, significantly increases costs from a societal perspective [27], which is broader than the patient- and household-level perspective used in our study. Cost-of-illness estimates restricted to paid work therefore exclude a component that, in our cohort, represented the entire indirect economic burden among patients outside the labor force.

Household production loss differed significantly according to the patient’s employment status and sex. In our research, the median household production loss for patients outside the labor force (retired, housewives, unemployed) was 679 Int$, whereas this value was 243 Int$ among actively working patients. Similarly, the median household production loss for female patients (679 Int$) was twofold higher than that of male patients (340 Int$). This discrepancy can be attributed to the influence of family structure and traditional gender roles in Türkiye on the socioeconomic epidemiology of ACS. Due to biological and hormonal protective mechanisms, ACS generally presents approximately ten years later in women than in men [28]. Women also tend to have a higher burden of baseline cardiovascular risk factors, such as hypertension, obesity, and diabetes mellitus, at the time of hospital admission [29]. Female patients experiencing myocardial infarction at an advanced age and with a higher burden of comorbidities may be more dependent on others for activities of daily living during the post-discharge period [30]. In traditional family structures, such as in Türkiye, women predominantly undertake the majority of unpaid domestic tasks [31]. Consequently, when female patients experience ACS, their inability to perform these routine chores may require substantially more assistance from others than is required by male patients. This division of domestic work is consistent with the greater number of assistance hours recorded for female patients and hence with their higher household production loss.

### 4.3. Indirect Economic Reflections of Household Composition and Social Isolation

The bivariate analyses of our study showed that patients living alone carried a higher economic burden than those living with a spouse. This association is unadjusted: household structure was not retained in the multivariate model, and living alone is strongly patterned by age and labor force participation in this cohort. The observed difference should therefore be interpreted as a marker of a broader socioeconomic configuration rather than as an independent driver of indirect cost. Therefore, living without familial support is associated with a higher short-term economic burden at the patient and household level. Household support has been associated with better treatment adherence and shorter recovery after a cardiac event [32], whereas patients living without such support report a longer time to return to work [23]. In this cohort the difference between household structures was confined to total indirect cost; neither absenteeism nor presenteeism differed significantly across the three groups, so the mechanism cannot be located in a specific cost component with these data. The presence of a spouse, however, may redistribute the household economic burden rather than eliminate it. Mazanec et al. reported that employed informal caregivers of patients with advanced disease lost close to a quarter of their work productivity, and those married caregivers experienced greater productivity loss than unmarried ones [33]. Our estimate values the assistance hours provided within the household at a replacement-cost shadow price, but it does not capture the paid work that the assisting person may have forgone; therefore, the reported figures should be interpreted as a lower bound of the total burden borne by the household.

### 4.4. The Impact of Sociodemographic Factors on Indirect Costs

Age and sex are core components of the GRACE algorithm, but formal collinearity diagnostics showed no problematic overlap. Both demographic variables were therefore entered into the Gamma generalized linear model alongside the GRACE score. Furthermore, the model’s largest association with indirect cost was occupational status. This is a structural feature of the Human Capital Approach rather than an independent risk relationship. Because productivity loss is calculated from paid work time, labor force position inherently enters the cost formula. The coefficient should therefore not be read as an estimate of the independent effect of occupation on cost, but rather as a description of how cost varies across occupational strata. No interaction terms were specified; the sex and occupational status coefficients are therefore main effects, estimated with the remaining covariates held constant.

Age and sex are widely recognized as primary determinants of productivity loss in the health economics literature. Although previous studies, such as Zhao and Winget [22], associated female sex with lower indirect cost burdens in bivariate analyses due to lower baseline labor force participation, our multivariable model identified a significant independent association between female sex and higher total indirect cost. No such difference was apparent in the unadjusted comparison (Table 3): women in this cohort were predominantly outside the labor force, where the absence of an absenteeism component lowers the unadjusted total. Consequently, the effect of their higher household production loss became visible only after occupational status was held constant. This pronounced household production loss aligns with evidence indicating that female patients with ACS typically present at an older age and carry a greater comorbid burden [34], factors that inherently increase the need for domestic assistance during recovery.

Educational attainment and socioeconomic position have been associated with both the recovery trajectory and health-related quality of life after a cardiac event [35]. The positive correlation between one-month household income and absenteeism costs partly reflects the wage-based framework of the Human Capital Approach, which values lost paid work time according to individual earnings. While household income is not a direct input in this calculation, its close alignment with patient wages means that income and employment status function as structural components of the cost formula rather than independent clinical predictors. Beyond this mathematical coupling, a weak inverse association was observed between household income and household production loss, indicating that lower-income households may experience a disproportionate loss of unpaid domestic labor. Such socioeconomic vulnerability is underscored by our finding that patients educated to primary school level or below experienced significantly higher household production losses than those with higher educational attainment. The difficulties faced by patients in these socioeconomically disadvantaged groups due to financial constraints regarding treatment adherence, maintaining appropriate nutritional conditions, and accessing professional care services may prolong recovery and increase household opportunity costs. Findings from Mahesh et al. and related literature demonstrate that socioeconomic disadvantages are among the primary barriers complicating disease management [36].

Another noteworthy finding regarding the complex interaction of sociodemographic and behavioral characteristics on costs was observed for smoking status. Bivariate analyses revealed that absenteeism costs for smoking patients were significantly higher than those for non-smokers, whereas household production loss was found to be higher in the non-smoking group. This can be explained by the fact that smoking advances the age of onset for acute ischemic events, particularly among young male patients in the active labor force, thereby directly driving absenteeism costs to their highest levels [37]. In contrast, the non-smoking group predominantly comprised elderly female patients with comorbidities such as diabetes and a more pronounced need for an attendant, making household production loss more prominent in this group. The two components did not offset each other: the excess absenteeism cost in smokers outweighed the excess household production loss in non-smokers, and total indirect cost was significantly higher in smokers. This aligns with the underlying mechanism, whereby an earlier disease onset among smokers corresponds to a higher rate of active labor force participation at the time of the index event, thereby shifting a greater proportion of productivity loss toward forgone wages. In the literature, the higher cost burden among the non-smoking or former smoker group is associated with the phenomenon of “reverse causality.” According to this view, patients often tend to quit smoking after experiencing a major clinical event such as myocardial infarction; this situation paradoxically results in a heavier chronic disease burden among non-smokers and, consequently, higher household production loss [38]. In our study, the comorbidities concentrated within the non-smoking group and the increased need for an attendant are consistent with this medico-economic dynamic.

### 4.5. The Impact of Clinical Characteristics and Prognostic Risk Scores on Indirect Costs

When the relationship between clinical severity and economic burden is evaluated within the context of health economics, a paradoxical picture emerges. The general consensus in the literature that costs increase in parallel with disease severity remains valid for direct medical costs such as expenditures for medication, intensive care, and surgery; however, this dynamic can be completely reversed for indirect costs [39].

In the bivariate analyses of our study, this pattern was evident for both chronic comorbidities and prognostic risk scores. Patients with diabetes and those with a history of previous coronary artery disease had significantly lower total indirect costs than those without these conditions, and patients with a GRACE score above 140 had lower costs than those below this threshold. Notably, this counterintuitive pattern was not limited to total costs but was also evident in absenteeism costs among the actively employed subgroup. The pattern was not monotonic across SCORE2 categories; in pairwise comparisons, a significant difference was observed exclusively between the high-risk and very-high-risk groups. Specifically, the very-high-risk group, characterized by the highest level of labor market withdrawal, exhibited lower indirect costs.

Contrary to expectations, the decrease in costs as the clinical picture worsens can be explained by the labor-market orientation of the Human Capital Approach and the typical sociodemographic structure of these risk groups. In the SCORE2 and GRACE algorithms, the primary parameter that increases the total score the most is age [40]. Consequently, very high-risk cases or individuals who have been followed for many years with diabetes/CAD constitute an elderly cohort that has already withdrawn from the labor market and retired, owing to the progressive nature of the disease and their advanced age. Since these patients are outside the active production process, the number of days lost from paid work during hospitalization due to ACS is mathematically considered zero, leading to lower estimated total indirect costs. Additionally, the paradoxical trend observed in absenteeism costs within the actively employed subgroup suggests that even among active workers, higher clinical severity (GRACE > 140) does not uniformly translate into higher measurable productivity losses under the Human Capital Approach, potentially reflecting baseline variations in income or occupational demands.

Gamma generalized linear modeling accounted for the bias inherent to bivariate analyses. In the multivariate model, the GRACE score retained no independent association with total indirect cost. SCORE2 was not entered into the model: it was developed for primary prevention in apparently healthy individuals without established atherosclerotic cardiovascular disease [17], age is its dominant input, and it is undefined below 40 years of age. Its bivariate association with cost is reported descriptively in Table 6. This finding indicates that the apparent cost variations across the cohort were largely structural differences related to labor force participation among healthier patients, rather than a direct economic impact of clinical severity itself. This finding is consistent with the literature emphasizing the importance of the working population aged 18–64 in determining the true socioeconomic burden of cardiovascular events. Research indicates that morbidity-related losses within the active labor force exceed even the costs of premature mortality [41]. Consequently, incorporating productivity losses into analyses is indispensable for ensuring accurate resource allocation in health policies [42].

Patients with a GRACE score below 140 or those classified as low/moderate risk in the SCORE2 assessment typically consist of individuals in the 40–55 age range who have high income levels and are active members of the labor force. Even a relatively mild ACS episode in this group leads to patients being absent from the workforce for weeks due to the hospitalization process and subsequent mandatory medical leave. The workplace productivity loss generated by a young and active employee’s absence during this period is substantial [43].

Literature links a high GRACE score exceeding 140 with an increased mortality risk and the requirement for complex clinical interventions, such as percutaneous coronary intervention (PCI) or coronary artery bypass grafting (CABG) [44]. Invasive interventions, particularly bypass surgery, significantly increase both direct medical costs and individual-level labor force loss due to prolonged recovery periods when compared to medical therapy [24]. Patient-based individual evaluations confirm that high-risk cases represent a higher burden not only clinically but also economically. However, in hospital-based analyses such as ours, the fact that a large portion of patients in the high-risk group remains outside the labor market due to their age shifts the statistical weight of total productivity loss costs toward the young and low-risk group of active workers.

It is crucial to acknowledge that the paradoxically lower indirect costs calculated for the ‘out of labor force’ cohort, particularly among those with high-risk clinical profiles, do not imply an actual absence of economic burden. Rather, this reflects a critical socioeconomic transition where severe ACS events force previously active individuals into early retirement or long-term disability. As highlighted in the literature, the sudden exit from the workforce due to cardiovascular events represents a permanent cessation of productivity, shifting the economic burden from measurable short-term absenteeism to a permanent lifetime income loss [45]. In our cohort, five patients who were actively employed prior to their ACS event had to take early retirement due to permanent loss of labor capacity. While these cases were excluded from our short-term one-month GLM models to maintain methodological homogeneity, their situation illustrates a form of loss that a 30-day measurement window cannot capture. Costs arising from permanent withdrawal from employment fall outside the scope of this study and would require longitudinal follow-up.

### 4.6. The Interaction Between Health-Related Quality of Life and Economic Burden

In the study, patients’ disease-specific health-related quality of life (HRQoL) was evaluated using the Myocardial Infarction Dimensional Assessment Scale (MIDAS), an instrument with internationally proven validity and reliability. Spearman correlation analysis showed that poorer disease-specific quality of life, indicated by a higher MIDAS score, was weakly associated with greater household production loss. Although bivariate analyses indicated a significant correlation between MIDAS scores and household production loss, MIDAS lost its independent significance for total indirect costs in the multivariate gamma model after adjusting for employment status and sex. A sensitivity analysis excluding five influential observations produced a nominally significant MIDAS coefficient; because this depends on the removal of a small number of observations, we do not interpret it as evidence of an independent association. This suggests that the economic impact of deteriorating quality of life is heavily contingent upon active workforce participation.

The observational design and the single one-month window of these data preclude establishing the direction of the association between quality of life and indirect cost. Physical limitations, such as functional impairment and exertional dyspnea developing after myocardial infarction, are associated with reduced health-related quality of life after myocardial infarction [46]. Poor quality of life restricts the patient’s ability to perform daily routines independently; consequently, this is reflected as a higher rate of domestic incapacitation and household production loss [47]. This study’s findings on labor and household production loss point to a phenomenon known as financial toxicity. It is defined as the combination of two factors: the financial cost of medical treatment and the mental stress an individual feels because of those expenses [48]. This process is not limited to merely creating a financial deficit for patients and their households. The financial stress triggered by illness-related income losses and out-of-pocket expenditures can act as a psychological barrier that compromises the patient’s adherence to diet and exercise programs, as well as secondary prevention regimens maintained through medication [49]. Although the multivariate model demonstrated that MIDAS is not independently associated with total indirect economic costs after adjusting for employment status, its bivariate relationship with increased household production loss is retained as a descriptive finding whose direction is consistent with greater functional limitation requiring more assistance. Whether this economic strain in turn affects adherence to secondary prevention was not measured in this study and remains a hypothesis for longitudinal work.

### 4.7. Limitations

When evaluating the research findings, structural limitations arising from the study design and the chosen health economics methodology must be taken into account.

The fact that the study was conducted as a single-center study at a tertiary education and research hospital in İzmir constitutes a limitation to the generalizability of the findings across Türkiye. This design may not fully reflect the socioeconomic and cultural diversity of different regions where agriculture or heavy industry is concentrated. Additionally, the participants’ education level—with 64.4% having a primary school education or below—and the characteristics of the income distribution were fundamental determinants in shaping the calculated monetary loss data. Accordingly, the cost estimates reported here describe the patients treated at this single center and should not be read as national estimates for Türkiye, or as reflections of the total societal burden of ACS, as direct medical expenditures, employer-side friction costs, and broader health-system costs fall outside the patient and household perspective adopted in this study. Extrapolation to the wider population would require nationally representative sampling and appropriate population weighting.

A second limitation is the relatively short follow-up duration. Because the follow-up period was restricted to the first month post-discharge, our study primarily captures the indirect costs associated with the acute cardiovascular event and the early convalescent phase. Although the iMTA Productivity Cost Questionnaire appropriately assesses short-term labor force and household production losses over a four-week retrospective period, this limited timeframe fails to capture the long-term economic impacts of acute coronary syndrome. As a result, productivity losses arising from late-stage complications, long-term disability, early retirement, or recurrent hospitalizations over a longer horizon are not reflected in our calculations, inevitably leading to an underestimation of the true long-term indirect cost burden. Additionally, because the iPCQ utilizes a strict 4-week recall period, our measurement of household production loss was confined to the post-discharge convalescence phase. Some household production loss during the acute inpatient stay was therefore not captured, as retrospective measurement would introduce recall bias. Our estimates for unpaid labor loss should be interpreted as a conservative lower bound.

The third limitation stems from the theoretical nature of the Human Capital Approach (HCA). This approach is based on the “full employment” model, which assumes that an employee who passes away or becomes permanently disabled cannot be replaced in the labor market and that all potential value the individual would have produced until retirement age constitutes a total societal loss. If the Friction Cost Approach (FCA) had been applied in the analysis, it would have been possible to obtain likely lower, yet more realistic cost data from an employer’s perspective, limited to the “friction period” required to recruit and train a replacement employee. Furthermore, structural limitations encountered during the statistical modeling phase should be emphasized. Total indirect cost was modelled with a Gamma GLM because it was strictly positive across the analytical cohort. The individual cost components could not be modelled in the same framework: absenteeism and presenteeism are undefined for patients outside the labor force. Specifically, presenteeism could be analyzed only in the 58 patients in salaried employment before the event, of whom 27 reported no on-the-job productivity loss. Given the size and zero-inflation of this subgroup, multivariable modelling of the sub-components would have been underpowered, restricting their evaluation to bivariate analyses. However, bivariate comparisons for this component were also underpowered, and the absence of significant differences should not be read as evidence of no difference.

Fourth, a notable methodological limitation of this study stems from the high-inflation environment in Türkiye during the data collection period. In economies marked by rapid currency devaluation, calculating indirect costs using nominal exchange rates can substantially underestimate the true domestic socioeconomic burden. To account for local purchasing power, the OECD Purchasing Power Parity (PPP) methodology was applied. Nevertheless, the choice of conversion metric inherently impacts cost estimates in volatile financial markets. For example, converting the median total indirect cost using the mean nominal exchange rate for 2025 (39.55 TRY/USD [50]) yields approximately 412 USD, compared with 1007 Int$ when PPP-adjusted. This divergence underscores a structural challenge in health economic evaluations within high-inflation settings, as our findings reflect domestic purchasing-power equivalents rather than nominal foreign-currency values.

Fifth, as individual interview dates were not retained, costs could not be deflated to a uniform price date at the patient level; the pooled estimates thus reflect the average price level across the March–August 2025 collection window, with the potential impact of this approximation bounded in Appendix A Table A2 [51]. Furthermore, while the questionnaire captured whether assistance was received, it did not record the provider’s relationship to the patient, precluding the differentiation between paid domestic help and informal family care; all assistance hours were therefore valued using the uniform replacement-cost shadow price.

Sixth, as detailed in the methods section, patients who passed away during the follow-up period or those who took early retirement due to permanent disability following ACS were excluded from the total indirect cost analysis. This approach was taken to maintain the dynamics of short-term measurement and to prevent biases that would be created by extreme outliers in the analysis. However, in the health economics literature, costs arising from mortality and permanent morbidity represent the severe economic burden a disease creates at the societal level. While the Human Capital Approach theoretically overestimates unit costs compared to the Friction Cost Approach, our short-term one-month measurement window and the exclusion of permanent disability cases mean the median 1007 Int$ figure likely represents a conservative lower threshold of the true longitudinal lifetime burden. Beyond these economic modeling constraints, specific clinical classifications were also restricted. Indirect costs were not stratified by ACS presentation type. The unstable angina subgroup comprised only seven patients, and any comparison across STEMI, NSTEMI, and unstable angina would have been underpowered. Furthermore, while SCORE2 is inherently designed for primary prevention in apparently healthy individuals, it was retrospectively utilized in this study as a proxy to estimate patients’ pre-event baseline cardiovascular risk; this constitutes a methodological limitation.

Seventh, the white-collar stratum comprised only nine patients within the analytical cohort of 140. Although this group differed from the out-of-labor-force reference in the multivariate model, the estimate is imprecise (cost ratio 4.785; 95% CI 3.104–7.375), and the two occupational strata were not statistically distinguishable from one another in pairwise comparison; estimates for this stratum should therefore be interpreted with caution. Bivariate comparisons across sociodemographic and clinical strata were exploratory and were not corrected for multiple testing; individual *p*-values close to the conventional threshold should therefore be interpreted conservatively.

Finally, a methodological limitation regarding the interpretation of predictors is the mathematical endogeneity between employment status, household income, and total indirect costs. Given that absenteeism and presenteeism costs are derived directly from patient wages, the observed strong associations are, in part, mathematical artifacts inherent to the Human Capital Approach. These variables should be interpreted cautiously as structural components of the cost formulation rather than purely independent epidemiological risk factors. Occupational status functions as a stratifying variable in this model, delineating how indirect costs vary across labor force strata rather than identifying occupation as an independent causal driver of those differences.

## 5. Conclusions

This study demonstrates that acute coronary syndrome generates a substantial short-term economic burden during the immediate out-of-hospital recovery phase from a patient and household perspective, with a median indirect cost of 1007 Int$ per patient observed in this single-center cohort. The multivariate generalized linear model indicates that occupational status and female sex were the factors most strongly independently associated with the indirect economic burden. Notably, after adjustment for occupational group and sex, clinical severity and prognostic risk scores no longer retain an independent statistical association. The higher indirect costs observed among white-collar and blue-collar workers relative to patients outside the labor force reflect both the wage-based structure of the Human Capital Approach and the concentration of measurable productivity loss within the employed subgroup.

## Figures and Tables

**Table 1:** Distribution of Sociodemographic and Cardiovascular Risk Factors of the Patient Group.

Variable	Category	*n*	%
Sex	Female	44	30.1
	Male	102	69.9
Salaried Employment Status	Yes	63	43.2
	No	83	56.8
Educational Status	Primary School and Below	94	64.4
	High School	27	18.5
	Higher Education	25	17.1
Social Security	General Health Insurance	122	83.6
	Green Card	15	10.3
	None	9	6.2
Household Structure	Spouse Supported (Nuclear Family)	63	43.2
	Living Alone	47	32.2
	Other Supported (Extended Family/Caregiver)	36	24.7
Occupational Status	Actively Working (Blue-collar)	53	36.3
	Actively Working (White-collar)	10	6.8
	Out of Labor Force	83	56.8
Smoking Status	Yes	93	63.7
	No	53	36.3
Diabetes	Yes	48	32.9
	No	98	67.1
Previous Coronary Artery Disease	Yes	41	28.1
	No	105	71.9
Familial Hypercholesterolemia	Yes	19	13.0
	No	127	87.0
Renal Failure	Yes	16	11.0
	No	130	89.0
History of Bypass	Yes	2	1.4
	No	144	98.6
Clinical Presentation	STEMI	80	54.8
	NSTEMI	59	40.4
	Unstable Angina	7	4.8
SCORE2 Risk Group *	Low	23	16.4
	Moderate	25	17.9
	High	17	12.1
	Very High	75	53.6
GRACE Risk Category	Normal Risk	86	58.9
	High Risk	60	41.1

Note: The “Out of Labor Force” category (*n* = 83) consists of retirees (*n* = 54), homemakers (*n* = 25), and unemployed individuals (*n* = 4). * SCORE2 was calculated only for the 140 patients aged 40 years and over, since the algorithm is undefined below this age. Percentages in this row are therefore based on *n* = 140 rather than on the full cohort of 146.

**Table 2:** Distribution of Baseline Characteristics, Prognostic Risk Scores, and Cost Parameters of the Patients.

Panel A. Clinical and Demographic Parameters					
Variables	*n*	Mean ± SD/Median (25th–75th Percentile)		Min–Max	Bootstrapped 95% CI for Mean
Patient Age (Years)	146	61 ± 12		33–93	58–63
Female	44	64 ± 14		33–93	59–68
Male	102	59 ± 11		35–83	57–62
Number of People Residing in Household	146	2 (2–4)		1–7	2.6–3.0
Length of Hospital Stay (Days)	146	4 (3–5)		2–50	4.2–4.9
MIDAS Total Score	146	19.3 (4.3–38.9)		0–79	20.2–27.3
GRACE Total Score	146	132 (116–155)		65–250	130–141
**Panel B. Indirect Cost Parameters**					
Variables	*n*	Mean ± SD/ Median (25th–75th Percentile) (TRY)	Mean ± SD/ Median (25th–75th Percentile) (Int$)	Min–Max (Int$)	Bootstrapped 95% CI for Mean (Int$)
Household Production Loss	140	8840 ± 7238/ 5501 (2751–11,003)	546 ± 447/ 340 (170–679)	0–1358	466–625
Absenteeism Cost	58	26,911 ± 19,325/ 23,483 (13,000–36,000)	1661 ± 1193/ 1450 (802–2222)	140–6379	1390–1951
Presenteeism Cost	58	6978 ± 9521/ 1550 (0–10,625)	431 ± 588/ 96 (0–656)	0–1975	297–562
Total Indirect Cost	140	22,880 ± 21,696/ 16,308 (5501–24,964)	1412 ± 1339/ 1007 (340–1541)	85–6470	1190–1643

Note: Patient age was normally distributed (Shapiro–Wilk *p* = 0.331) and is therefore presented as mean ± SD in both the text and this table. Cost parameters were right-skewed and are primarily described by medians; means and standard deviations are reported concurrently to facilitate aggregate economic burden estimation. The variations in sample sizes (*n*) across parameters reflect specific methodological sub-cohorts: (1) *n* = 146 represents the total initial study cohort for baseline sociodemographic and clinical characteristics. (2) *n* = 140 represents the cohort utilized for household production loss and total indirect costs; a total of 6 patients (1 death and 5 early retirements due to permanent disability) were excluded from this calculation as their permanent labor loss is incompatible with the short-term measurement framework of the iPCQ. (3) *n* = 58 is used for labor productivity loss parameters (absenteeism and presenteeism costs), which were calculated exclusively for the subgroup of patients in active salaried employment prior to the ACS event, since these components are undefined for patients with no paid work time to forgo. Bootstrapped 95% Confidence Intervals (CIs) for the mean are based on 1000 bootstrap samples (Bias-corrected and accelerated).

**Table 3:** Comparison of Indirect Costs According to Sociodemographic and Clinical Variables.

Independent Variables	Household Production Loss (Int$) (*n* = 140)	Absenteeism Cost (Int$) (*n* = 58)	Presenteeism Cost (Int$) (*n* = 58)	Total Indirect Cost (Int$) (*n* = 140)
Sex				
Female	679 (230–1358)	3035 (1430–4604)	639 (0–1312)	1358 (340–1358)
Male	340 (170–679)	1307 (802–2052)	62 (0–560)	947 (340–2071)
*p*-value	**<0.001**	**0.041**	0.264	0.725
Salaried Employment Status				
No	679 (315–1358)	N/A	N/A	679 (315–1358)
Yes	243 (121–485)	1450 (802–2222)	96 (0–656)	2113 (994–3580)
*p*-value	**<0.001**	N/A	N/A	**<0.001**
Smoking Status				
No	679 (170–1358)	669 (165–1656)	31 (0–497)	819 (340–1358)
Yes	340 (170–679)	1646 (823–2299)	99 (0–725)	1075 (679–2562)
*p*-value	**0.019**	**0.031**	0.634	**0.007**
GRACE Risk Category				
Normal Risk (<140)	340 (170–679)	1646 (823–2413)	216 (0–793)	1358 (679–2789)
High Risk (≥140)	340 (170–1358)	840 (473–1656)	0 (0–438)	679 (340–1358)
*p*-value	0.364	**0.023**	0.302	**0.001**
Presence of Diabetes				
No	340 (170–679)	1564 (827–2338)	194 (0–748)	1224 (679–2451)
Yes	340 (176–1358)	813 (543–1883)	0 (0–441)	679 (340–1358)
*p*-value	0.270	0.097	0.283	**0.005**
Previous Coronary Artery Dis.				
No	340 (170–679)	1564 (823–2261)	194 (0–782)	1155 (679–2015)
Yes	340 (170–898)	1132 (540–1962)	0 (0–500)	679 (340–1358)
*p*-value	0.989	0.270	0.253	**0.032**
Renal Failure				
No	340 (170–679)	1564 (823–2261)	136 (0–702)	982 (340–1955)
Yes	849 (188–1358)	561 (237–1001)	15 (0–429)	1358 (364–1358)
*p*-value	0.112	**0.022**	0.539	0.703
Familial Hypercholesterolemia				
No	340 (170–679)	1451 (802–2222)	173 (0–679)	1019 (389–1861)
Yes	340 (170–1189)	1152 (658–1728)	0 (0–556)	993 (340–1358)
*p*-value	0.922	0.390	0.339	0.608

Note: Monetary cost values in the table are presented as median (25th–75th percentile). *p*-values were calculated using the Mann–Whitney U test due to the non-normal distribution of the data. N/A: not applicable—absenteeism and presenteeism are undefined for patients outside the labor force. Bold indicates statistical significance at *p* < 0.05.

**Table 4:** Comparison of Indirect Costs According to Occupational Group.

Independent Variable (Occupational Group)	Household Production Loss (Int$) (*n* = 140)	Absenteeism Cost (Int$) (*n* = 58)	Presenteeism Cost (Int$) (*n* = 58)	Total Indirect Cost (Int$) (*n* = 140)
Active Worker (Blue-collar) ***(n*** = 49)	243 (146–485)	1296 (792–2114)	173 (0–725)	1901 (959–3495)
Active Worker (White-collar) (***n*** = 9)	170 (106–291)	2068 (1137–3426)	0 (0–434)	2407 (1483–4189)
Out of Labor Force (***n*** = 82)	679 (315–1358)	N/A	N/A	679 (315–1358)
Test statistic	24.664	295.000	162.500	56.467
*p*-value	**<0.001**	0.110	0.189	**<0.001**
Significant Difference (Post Hoc)	Out of Labor Force > Blue-collar (adj. ***p* < 0.001**); Out of Labor Force > White-collar (adj. ***p* = 0.001**)	-	-	Blue-collar > Out of Labor Force (adj. ***p* < 0.001**) White-collar > Out of Labor Force (adj. ***p* < 0.001**)

Note: Cost values are presented as median (25th–75th percentile). Household production loss and total indirect cost were compared across the three occupational groups using the Kruskal–Wallis test (df = 2), with pairwise comparisons performed using Dunn’s test with Bonferroni adjustment. Absenteeism and presenteeism were analyzed exclusively within the actively employed subgroup and were therefore compared between the two working groups using the Mann–Whitney U test; these parameters are not applicable (N/A) to patients outside the labor force. Blue-collar and white-collar workers did not differ significantly from each other in household production loss (adj. *p* = 0.526) or total indirect cost (adj. *p* = 1.000). N/A: not applicable—absenteeism and presenteeism are undefined for patients outside the labor force. Bold indicates statistical significance at *p* < 0.05.

**Table 5:** Comparison of Indirect Costs According to Household Structure.

Independent Variable (Household Structure)	Household Production Loss (Int$) (*n* = 140)	Absenteeism Cost (Int$) (*n* = 58)	Presenteeism Cost (Int$) (*n* = 58)	Total Indirect Cost (Int$) (*n* = 140)
Spouse Supported (Nuclear Family)	340 (170–982)	1352 (712–2752)	0 (0–355)	679 (340–1358)
Living Alone	352 (182–679)	1811 (808–2338)	401 (0–748)	1358 (679–2505)
Other Supported (Extended Family, etc.)	340 (170–679)	1204 (772–1852)	86 (0–625)	1137 (679–1913)
Test statistic	0.473	1.336	3.365	7.617
*p*-value	0.790	0.513	0.186	**0.022**
Significant Difference (Post Hoc)	-	-	-	Living Alone > Spouse Supp. (adj. *p* = 0.031)

Note: Cost values are presented as median (25th–75th percentile). Groups were compared using the Kruskal–Wallis test (df = 2); pairwise comparisons were performed using Dunn’s test with Bonferroni adjustment only where the overall test was significant. Remaining pairwise comparisons for total indirect cost: other supported vs. spouse supported, adjusted *p* = 0.166; other supported vs. living alone, adjusted *p* = 1.000. Presenteeism cost contains a high proportion of zero values (27/58), which reduces the power of the comparison. Absenteeism and presenteeism were analyzed exclusively within the actively employed subgroup. Bold indicates statistical significance at *p* < 0.05.

**Table 6:** Comparison of Cost Indicators According to SCORE2 Risk Group.

Independent Variable (SCORE2 Risk Group)	Household Production Loss (Int$) (*n* = 135)	Absenteeism Cost (Int$) (*n* = 55)	Presenteeism Cost (Int$) (*n* = 55)	Total Indirect Cost (Int$) (*n* = 135)
Low Risk	340 (170–679)	2006 (1008–2752)	448 (0–773)	1343 (340–3111)
Moderate Risk	340 (170–679)	1173 (772–1968)	96 (0–826)	1121 (679–1821)
High Risk	509 (170–679)	1296 (840–2222)	0 (0–833)	1406 (1013–3002)
Very High Risk	340 (170–1140)	1132 (625–1998)	0 (0–548)	679 (340–1358)
Test statistic	2.120	3.129	1.485	13.035
*p*-value	0.548	0.372	0.686	**0.005**
Significant Difference (Post Hoc)	-	-	-	High > Very High (adj. ***p* = 0.010**)

Note: Cost values are presented as median (25th–75th percentile). Groups were compared using the Kruskal–Wallis test (df = 3); pairwise comparisons were performed using the Dunn test with Bonferroni adjustment only where the overall test was significant. All remaining pairwise comparisons for total indirect cost were non-significant (adjusted *p* ≥ 0.272). Denominators (*n* = 135 and *n* = 55) reflect the exclusion of patients under 40 years of age from the respective base cohorts (*n* = 140 and *n* = 58), as SCORE2 is not calculated below this age. Bold indicates statistical significance at *p* < 0.05.

**Table 7:** Spearman Correlation Coefficients Between Sociodemographic, Clinical, and Cost Variables.

Independent Variables	Household Production Loss (*n* = 140)	Absenteeism Cost (*n* = 58)	Presenteeism Cost (*n* = 58)	Total Indirect Cost (*n* = 140)
Patient Age	0.233 **	−0.344 **	−0.294 *	−0.378 **
One-month Household Income	−0.257 **	0.506 **	0.167	0.354 **
MIDAS Score	0.198 *	−0.105	0.119	−0.034
GRACE Score	0.086	−0.193	−0.084	−0.288 **

Note: Coefficients are Spearman’s rho. Household production loss and total indirect cost were analyzed in the full cost cohort (*n* = 140); absenteeism and presenteeism were analyzed exclusively within the actively employed subgroup (*n* = 58), consistent with Table 3, Table 4, Table 5 and Table 6. * Significant at *p* < 0.05. ** Significant at *p* < 0.01.

**Table 8:** GLM Analysis Results for Factors Associated with Total One-Month Indirect Costs.

Variables	Wald Chi-Square	*p*-Value	Cost Ratio [Exp(B)]	95% Confidence Interval (Lower–Upper)
Constant (Intercept)	3933.635	**<0.001**	561.77	460.93–684.67
Occupational Group (Overall Model Effect)	89.051	**<0.001**	-	-
- White-collar	77.427	**<0.001**	4.785	3.104–7.375
- Blue-collar	50.295	**<0.001**	4.109	2.999–5.629
- Out of Labor Force (Reference)	-	**-**	1.000	-
Sex (Overall Model Effect)	8.322	**0.004**	-	-
- Female	8.322	**0.004**	1.465	1.130–1.900
- Male (Reference)	-	-	1.000	-
Patient Age (Continuous-Centered)	0.147	0.701	1.002	0.990–1.015
GRACE Score (Continuous-Centered)	0.437	0.508	0.999	0.994–1.003
MIDAS Total Score (Continuous-Centered)	2.801	0.094	1.005	0.999–1.010

Note: Generalized linear model with a Gamma distribution and log-link function, estimated with Huber-White robust standard errors (*n* = 140). The scale parameter was estimated by maximum likelihood as 0.453. The model was significant against the intercept-only model (likelihood ratio χ^2^ = 96.392, df = 6, *p* < 0.001), and the scaled Pearson χ^2^/df ratio of 0.907 indicated adequate fit. A modified Park test yielded a variance-function power of 1.71 (95% CI 1.31–2.12), consistent with the Gamma family. Multicollinearity was not present (maximum VIF = 1.978, minimum tolerance = 0.506). Continuous covariates were centered at the analytical cohort means (age 60.92 years, GRACE 136.33, MIDAS 24.05); the intercept therefore represents the expected one-month indirect cost for a male patient outside the labor force at these baseline values. For continuous variables, the cost ratio represents the multiplicative effect of a one-unit increase. Bold indicates statistical significance at *p* < 0.05.

## Data Availability

The data presented in this study are available upon request from the corresponding author. The data are not publicly available due to privacy and ethical restrictions regarding the protection of personal health information of the participants.

## Abbreviations

The following abbreviations are used in this manuscript:

ACS	Acute Coronary Syndrome
CABG	Coronary Artery Bypass Grafting
CAD	Coronary Artery Disease
CI	Confidence Interval
CVD	Cardiovascular Disease
DALY	Disability-Adjusted Life Year
ESC	European Society of Cardiology
FCA	Friction Cost Approach
GBD	Global Burden of Disease
GLM	Generalized Linear Model
GRACE	Global Registry of Acute Coronary Events
GSS	General Health Insurance
HCA	Human Capital Approach
HIMS	Hospital Information Management System
HRQoL	Health-Related Quality of Life
iPCQ	iMTA Productivity Cost Questionnaire
MIDAS	Myocardial Infarction Dimensional Assessment Scale
NSTEMI	Non-ST-Segment Elevation Myocardial Infarction
OECD	Organisation for Economic Co-operation and Development
PCI	Percutaneous Coronary Intervention
PPP	Purchasing Power Parity
SCORE2	Systematic Coronary Risk Estimation 2
STEMI	ST-Segment Elevation Myocardial Infarction
UAP	Unstable Angina Pectoris
USD	US Dollars
VNRS	Verbal Numerical Rating Scale

## Appendix A

**Table A1:** Comparison of Indirect Costs According to Educational Status.

Independent Variable (Educational Status)	Household Production Loss (Int$) (*n* = 140)	Absenteeism Cost (Int$) (*n* = 58)	Presenteeism Cost (Int$) (*n* = 58)	Total Indirect Cost (Int$) (*n* = 140)
Primary School and Below	364 (182–1358)	1111 (761–2037)	173 (0–559)	1067 (340–1358)
High School	340 (170–679)	1590 (936–1998)	267 (0–1119)	995 (679–3364)
Higher Education	206 (103–594)	2160 (741–3344)	0 (0–849)	911 (389–2954)
Test statistic	9.359	3.353	0.808	1.388
*p*-value	**0.003**	0.187	0.667	0.499
Significant Difference (Post Hoc)	Primary School and Below > Higher Education (adj. ***p* = 0.009**)	-	-	-

Note: Cost values are presented as median (25th–75th percentile). Groups were compared using the Kruskal–Wallis test (df = 2); pairwise comparisons were performed using the Dunn test with Bonferroni adjustment where the overall test was significant. Absenteeism and presenteeism were analyzed exclusively within the actively employed subgroup. Bold indicates statistical significance at *p* < 0.05.

**Table A2:** Sensitivity analysis.

Scenario	Assumption Varied	Median Total İndirect Cost
Base case	Costs at 2025 prices (March–August 2025 collection window); OECD PPP for GDP 2025 = 16.20 TRY per Int$	TRY 16,308 (Int$ 1007)
S1	All costs rebased to the March 2025 price level (CPI factor 0.9491)	TRY 15,478 (Int$ 955)
S2	All costs rebased to the August 2025 price level (CPI factor 1.0478)	TRY 17,088 (Int$ 1055)
S3	Nominal market exchange rate instead of PPP (2025 mean: 39.55 TRY/USD)	TRY 16,308 (USD 412)

Note: Scenarios S1 and S2 bound the effect of intra-window inflation by rebasing all observations to the first and last month of the data collection period, using the monthly consumer price index retrieved from the Central Bank of the Republic of Turkey (CBRT) Electronic Data Delivery System. Rebasing multipliers were computed using a relative index constructed from the official monthly CPI changes reported for the March–August 2025 period (monthly changes: 3.00% in April, 1.53% in May, 1.37% in June, 2.06% in July, and 2.04% in August). Setting March 2025 as the base (100.00), the cumulative relative index reached 110.40 by August, with a 6-month mean of 105.36. The multipliers were calculated as the ratio of the target month to this mean (100.00/105.36 = 0.9491 for March; 110.40/105.36 = 1.0478 for August). Furthermore, International dollar values were obtained by dividing nominal TRY amounts directly by the 2025 OECD purchasing power parity factor for gross domestic product in Türkiye (16.20 TRY per Int$). No further adjustments were applied to the conversion metric. Because each scenario applies a single constant multiplier to every observation, ranks are preserved exactly; all Mann–Whitney U, Kruskal–Wallis and Spearman results, and all cost ratios of the generalized linear model, are numerically identical to the base case, and only the model intercept shifts by the logarithm of the multiplier. S3 varies the conversion metric rather than the price base. Sensitivity to the choice of shadow price was assessed separately: valuing assistance hours at the gross rather than the net minimum wage (TRY 26,005.50 vs. TRY 22,104.67) would raise the median household production loss from Int$ 340 to Int$ 399.
